# Treatment Effectiveness of Volar Plates in Distal Radius Dorsal Rim Fractures

**DOI:** 10.1055/s-0044-1790579

**Published:** 2024-12-21

**Authors:** Uğur Bezirgan, Erdinç Acar, Yener Yoğun, Merve Dursun Savran, Ömer Halit Keskin, Mehmet Armangil

**Affiliations:** 1 Departamento de Ortopedia, Traumatologia e Cirurgia da Mão, Faculdade de Medicina, Ankara University, Altındağ, Ankara, Turquia; 2 Departamento de Ortopedia, Traumatologia e Cirurgia da Mão, Ankara Bilkent City Hospital, Çankaya, Ankara, Turquia

**Keywords:** bone plates, fracture fixation, internal, radius fractures, retrospective studies

## Abstract

**Objective** To assess the efficacy of distal radius volar plates in cases involving dorsal fragments at the Ulnar Corner (UC) and Lister Tubercle (LT).

**Methods** A retrospective study that included patients with distal radius fractures (DRFs) featuring UC and LT dorsal fragments treated with volar plates. The exclusion criteria comprised lunate facet fractures, UC fragment ratio below 25%, and patients treated with dorsal plates. Radiographic and tomographic measurements included radial length (RL), radial inclination (RI), ulnar variance (UV), palmar tilt (PT), fragment areas, UC fragment ratio, and displacement. The scores on the Gartland Werley (GW) classification and on the Disabilities of the Arm, Shoulder, and Hand (DASH) questionnaire, as well as grip strength, and range of motion (ROM), were assessed.

**Results** The study involved 17 male and 5 female subjects (mean age: 39.7 ± 10.7 years). The UC and LT fragments displayed mean areas of 1.6 ± 0.7cm ^2^ and UC fragment ratio of 0.4 ± 0.1. The fixation rates for UC and LT fragments were of 18.2% and 31.8% respectively. Improved RI, UV, and PT were noted postoperatively. The mean GW and DASH scores were of 2.1 ± 2.0 and 4.3 ± 3.2 respectively. Grip strength on the operated side was of 89.5 ± 9.8% of the healthy side, and at least 90.9% of the patients achieved adequate ROM.

**Conclusion** While volar plates are the standard treatment for intra-articular DRFs, displaced dorsal fragments can impact the outcomes. Mini dorsal incisions may aid in the fixation of UC fragments that are challenging to secure with volar plates, preserving joint health.

## Introduction

Distal radius fractures (DRFs) are common and usually caused by high-energy trauma, such as an accidental fall or traffic accident. 1 High-energy injuries can lead to intra-articular comminuted DRFs, which often include free fracture fragments displaced either dorsally or volarly. Since these fractures are included in the intra-articular fracture classification, they require anatomic reduction and rigid fixation according to the principles of the Association of the Study of Internal Fixation (Arbeitsgemeinschaft für Osteosynthesefragen, AO, in German). 2

With the development of fixed-angle volar locking plates, these fractures are usually fixed only through a volar incision. 3 With this approach, free fragments can be easily seen and reduced. In contrast, direct control of dorsal fragments is impossible. Suppose the fixation of a dorsal fragment with the volar approach is not stable enough; the fracture may be displaced, leading to malunion and nonunion of the fracture, as well as posttraumatic arthritis and worsening of wrist function. 4 5 If the dorsal free fragments are large enough, they are fixed with screws in the distal holes of the volar plate. However, evaluating whether these screws firmly hold dorsal-free fragments through fluoroscopy in the operating room is not always possible.

The dorsal plate technique has also been described as a fixation method for dorsal intra-articular unstable fragments. Still, serious complications have been observed in dorsal plating, including tendon irritation and rupture. 6 Palmar and dorsal combined approaches are efficient in reduction and fixation, but this technique negatively affects the distal radius and skin vascularity with additional soft-tissue trauma. 7

The primary aim of the present study was to evaluate the effectiveness of volar plates in the fixation of distal radius dorsal rim fractures in promoting reduction and stabilization of the ulnar corner (UC) and Lister tubercle (LT) fragments.

## Materials and Methods

**Fig. 1:**
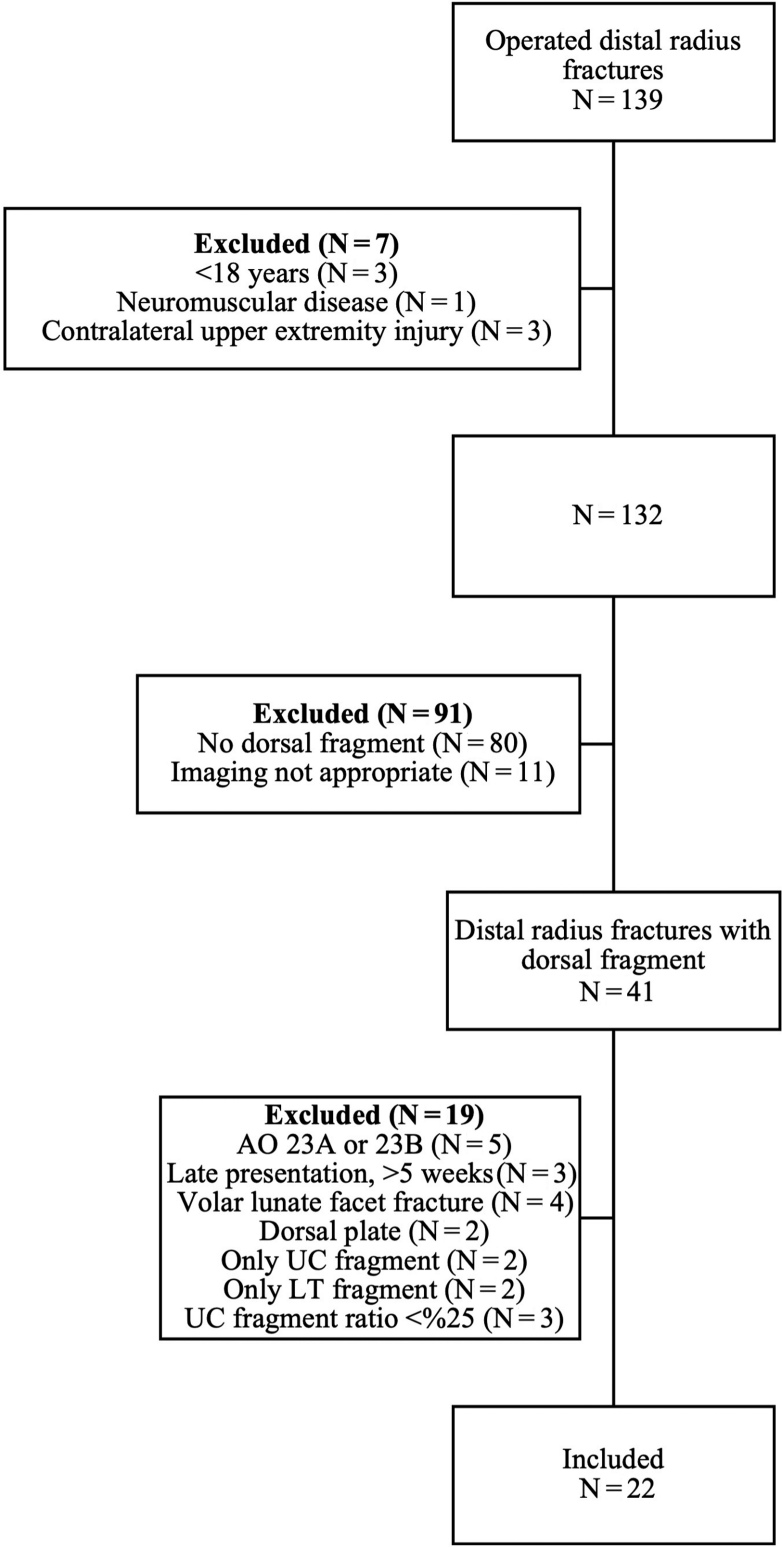
The process of inclusion and exclusion of patients.

The current retrospective cohort study of distal radius fractures with dorsal fragments was approved by the institutional Ethics Committee (under number E1-23-3412). Patients operated on for DRFs between May 2019 and October 2021 were found in the hospital records. Subjects with DRFs with no dorsal fragments, AO types 23A or B fractures, patients who presented later than 5 weeks after the fracture, those with volar lunate facet fractures, subjects operated on with dorsal plates, and patients with only UC or LT fragments were excluded. In our clinic, given that the DRFs were AO type C, routine X-rays and computed tomography (CT) scans were performed postoperatively, with particular emphasis on the long-term monitoring of complications, and interventions were carried out in certain cases. Patients lost to follow-up and with missing x-rays or CT scans were excluded. Patients with a UC fragment ratio lower than 25% were also excluded from the study. Subjects younger than 18 years of age, with neuromuscular diseases, and with contralateral upper extremity injuries were also excluded. We included 22 with intra-articular DRFs with UC and LT fragments, treated with a volar plate. The inclusion and exclusion of patients are detailed in Fig. 1 .

**Fig. 2:**
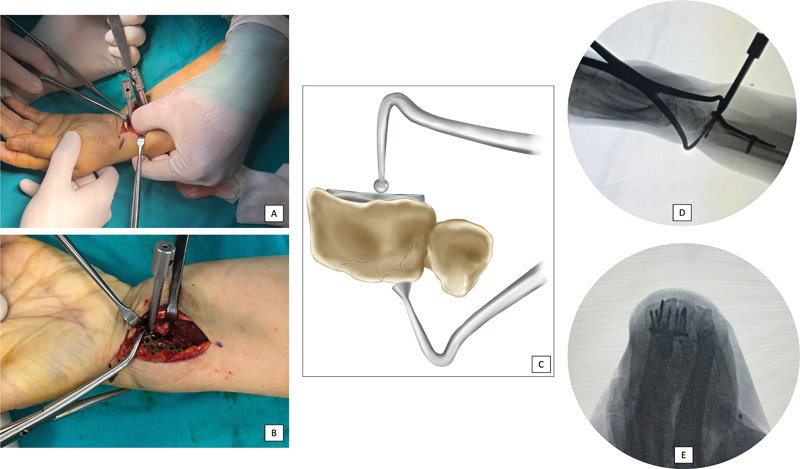
The 'trick' in using the volar plate to fix a dorsal ulnar corner (UC) fragment. Securing the plate with the thumb on the plate and index finger on the dorsum of the wrist ( **A** ). Securing the dorsal UC fragment with a broad Weber clamp ( **B,C** ). Drilling under fluoroscopic guidance (D). Controlling the screws for reduction of the radial notch and undesired screw penetration of the distal radioulnar joint (DRUJ) in a skyline view with fluoroscopy ( **E** ).

All of the surgeries were performed by the 2 orthopedic hand surgeons (UB and EA) with levels 3 to 4 of surgical experience according to the study by Tang and Giddins. 8 Using a volar approach and pronating the radius shaft, the dorsal large fragment was temporarily fixed with a Kirschner wire. A screw was then used to secure the fixation over the plate, ensuring that it did not penetrate the dorsal cortex to avoid extensor tendon rupture. Additionally, care was taken to ensure that the plate did not cross the watershed line, preventing flexor tendon rupture. Once temporary fixation with the Kirschner wire ensured plate screw fixation, it was removed, and if the fixation remained stable, the surgery was concluded. The most critical ‘trick’ in using the volar plate to fix the examined dorsal UC fragment involves securing the volar and dorsal UCs with a broad Weber clamp, followed by drilling under fluoroscopic guidance ( Fig. 2 ). A thorough understanding of the radial notch anatomy is necessary to accomplish this without perforating the joint. If the fragment is too small to be fixed with a screw, during drilling, the thumb and index finger of the left hand can act as a Weber clamp, aligning the fragments and enabling the careful placement of the screw over the plate. During surgery, a well-captured ‘skyline’ radiograph on fluoroscopy not only reveals the relationship of the most ulnar screw with the dorsal cortex but also enables visualization of its relationship with the radial notch. We primarily use this tangential view to visualize the radial notch during surgery because we never penetrate the dorsal cortex. Achieving this technique may not be straightforward for a surgeon with levels 1 to 2 of experience.

**Fig. 3:**
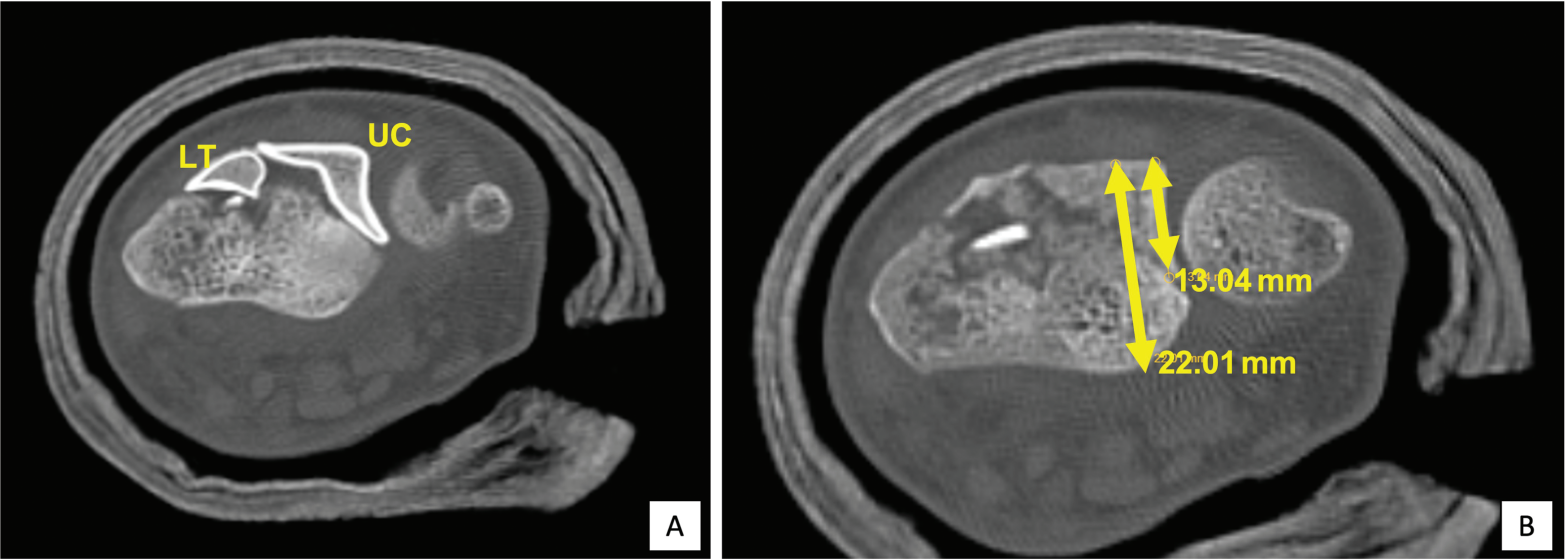
Illustration of the dorsal fragments ( **A** ). Measurement of the UC fragment ratio ( **B** ). **Abbreviations:** LT, Lister tubercle; UC, ulnar corner.

**Fig. 4:**
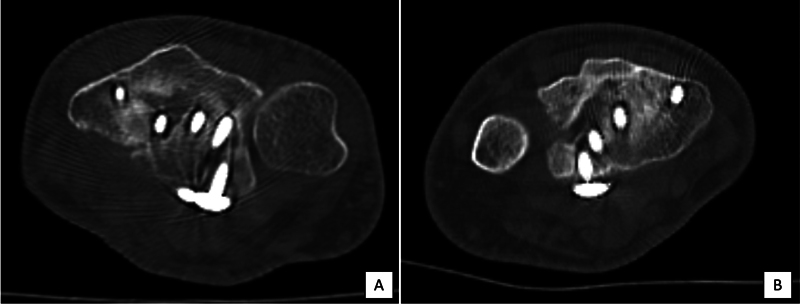
In this example, the dorsal fragment was fixated with a screw ( **A** ). In this other example, the dorsal fragment could not be fixed with a screw; union was achieved from the radial side ( **B** ).

Once the patients were identified from the hospital records, their pre- and postoperative radiologic studies, direct X-rays, and CT scans were investigated. As radiologic parameters, radial inclination (RI), radial height (RH), ulnar variance (UV), and palmar tilt (PT) were measured from pre- and postoperative X-rays of the injured side. Postoperative radiographs were taken at 6 weeks, 3 months, 6 months, and 1 year, and then, once a year apart. The first and last x-rays of the patients were evaluated. All measurements were performed by the senior surgeon (UB). The Jupiter 9 10 criteria were used for the radiologic variables. The unacceptance criteria were radial inclination < 10 ^o^ , volar tilt > 20 ^o^ or dorsal tilt > 20 ^o^ , radial height < 10 mm, ulnar variance > 2 mm, and intraarticular step or gap > 2 mm. 9 10 From the CT scans, the width and depth of the dorsal UC and LT fragments were measured separately, and the surface area was calculated. The total area of the dorsal fragment is the sum of the UC and LT fragments. Also, the ratio of the UC fragment depth to the distal radius depth was calculated ( Fig. 3 ). We also recorded whether or not the fragment was fixed with a screw from the volar plate ( Fig. 4 ). Moreover, the percentage of the area of the dorsal fragment that was fixed with a screw was calculated.

The patients underwent a follow-up visit for the functional evaluation, which included the scores on the Gartland Werley (GW) classification and on the Disabilities of the Arm, Shoulder, and Hand (DASH) questionnaire, grip strength measurement with a dynamometer, and range of motion (ROM) measurement with a goniometer. A junior surgeon calculated the scores, and a senior surgeon performed the measurements. The normal maximum ROM values were accepted as 70 ^o^ of extension, 75 ^o^ of flexion, 20 ^o^ of radial deviation, 35 ^o^ of ulnar deviation, 70 ^o^ of pronation, and 85 ^o^ of supination. 11 According to Ryu et al., 12 most of the functional tasks could be accomplished with 70% of the maximum wrist ROM, so the minimum accepted values required were of 49 ^o^ of extension, 52.5 ^o^ of flexion, 14 ^o^ of radial deviation, 24.5 ^o^ of ulnar deviation, 49 ^o^ of pronation, and 59.5 ^o^ of supination. Grip strength was measured for both the injured and the healthy sides, and its percentage on the injured compared to the healthy side was calculated for further analysis.

Descriptive and inferential analyses were performed. The Shapiro-Wilk test was used to assess normality. The Chi-squared and Fisher exact tests were used for the categorical variables. A *t* -test was performed to analyze the parametric data of the groups. The Mann Whitney U test was performed to analyze the non-parametric data of the groups. A paired t-test was used to compare dependent groups. Values of *p* lower than 0.05 were deemed significant. For the correlation analysis, the Pearson correlation test was used if both variables were parametric, whereas the Spearman correlation test was used if any of the variables were non-parametric. Rho ≥ 0.8 was accepted as a strong correlation, according to Chan. 13

## Results

The demographic data, dimensions of the fragments, functional scores, and ROM data are summarized in Table 1 . The mean follow-up was of 24.1 ± 4.1 (minimum 17; maximum: 32) months. At the end of the last follow-up, survey forms were filled out, and the functional scores were calculated. The mean grip strength on the operated side was of 35.0 ± 6. 0kg, whereas, on the healthy side, it was of 39.1 ± 4.6 kg, a significantly higher value ( *p* = 0.0002). The mean percentage of grip strength on the operated side compared to the healthy side was of 89.5 ± 9.8%. The minimum required ROM was achieved by 90.9% for flexion, extension, radial deviation and ulnar deviation, whereas all patients (100%) achived normal range for pronation and supination.

**Table 1:** Demographic, dorsal fragment and functional data of the study sample

**Age (years): mean ± SD**	**39.7 ± 10.7**
**Gender: n (%)** ** Female** ** Male**	5 (22.7) 17 (77.3)
**Side: n (%)** ** Right** ** Left** **Dominance: n (%)**	11 (50.0) 11 (50.0) 11. (50.0)
**Time until presentation (days): mean ± ** SD	15.6 ± 11.3
**Follow-up time (months): mean ± SD**	24.1 ± 4.1
**Ulnar corner** ** Width (mm): mean ± SD** ** Depth (mm): mean ± SD** ** Area (cm ^2^ ): mean ± SD ** ** Screw fixation of the fragment: ^a^ n (%) ** ** Ulnar corner fragment ratio: mean ± SD** ** Preoperative gap (mm): mean ± SD** ** Postoperative gap (mm): mean ± SD**	13.4 ± 3.1 6.6 ± 1.4 0.9 ± 0.3 4 (18.2) 0.4 ± 0.1 2.0 ± 1.2 0.2 ± 0.6
**Lister tubercle** ** Width (mm): mean ± SD** ** Depth (mm): mean ± SD** ** Area (cm ^2^ ): mean ± SD ** ** Screw fixation of the fragment: ^a^ n (%) **	9.3 ± 3.00 7.3 ± 3.2 0.7 ± 0.5 7 (31.8)
** Total area of the fragment (cm ^2^ ): mean ± SD ** **Area of the fixated fragment (%)**	1.6 ± 0.7 44.5 ± 41.3
**GW score: mean ± SD**	2.1 ± 2.0
**DASH score: mean ± SD**	4.3 ± 3.2
**Grip strength (kg): mean ± SD** ** Operated side** ** Healthy side (control)** **Operated/Control (%)**	35.0 ± 6.0 39.1 ± 4.6 89.5 ± 9.8
** Range of motion: mean ± SD; n (%) ^b^** ** Extension** ** Flexion** ** Radial deviation** ** Ulnar deviation** ** Pronation** ** Supination**	69.6 ± 9.3; 20 (90.9%) 70.5 ± 10.3; 20 (90.9%) 21.1 ± 4.4; 20 (90.9%) 28.5 ± 5.9; 20 (90.9%) 73.4 ± 6.6; 22 (100%) 78.5 ± 8.3; 22 (100%)

**Abbreviations:** DASH, Disabilities of the Arm, Shoulder and Hand questionnaire; GW, Gartland Werley classification; SD, standard deviation.

**Notes:** For the categorical variables, ‘n’ is the number of patients that fulfill the condition, and ‘%’ is the percentage of these patients. ^a^ Screw fixation of the fragment is the presence of a screw that holds the fragment. ^b^ In this context, ‘n’ is the number of patients who were able to achieve the minimum required range for each specific motion, and “%” is the percentage of these patients.

Table 2 presents a comparison of the pre- and postoperative radiologic parameters. The RH, UV, and PT improved significantly after the surgery. All dorsal fragments were displaced and successfully reduced with the volar surgical approach using the Orbay technique. Fixation was achieved via a screw from the volar plate in 4 patients for the UC fragment and in 7 patients for the LT fragment. However, an UC displacement of 2.4 mm was detected in one patient who did not comply with the follow-up protocols. Moreover, in 81.82% of the patients, all parameters were within the normal range.

**Table 2:** Pre- and postoperative radiological parameters of the study sample

Parameter	Preoperative: mean ± SD	Postoperative: mean ± SD	*p*	Difference (post and pre): mean ± SD	Preoperative normal range: n (%)	Postoperative normal range: n (%)	*p*
**Radial inclination**	22.6 ± 5.1	23.2 ± 3.1	0.5151	0.6 ± 4.4	22 (100%)	22 (100%)	1.000
**Radial height**	11.8 ± 3.6	13.1 ± 2.4	**0.0016**	1.3 ± 1.5	18 (81.8%)	20 (90.9%)	0.1573
**Ulnar variance**	1.3 ± 1.6	0.1 ± 1.6	**0.0124**	-1.2 ± 2.0	10 (45.5%)	18 (81.8%)	**0.0047**
**Palmar tilt**	-4.3 ± 15.8	3.8 ± 3.7	**0.0457**	9.1 ± 16.1	18 (81.8%)	22 (100%)	**0.0455**
**Fragment gap**	2.0 ± 1.2	0.2 ± 0.6	**0.0000**	1.8 ± 0.8	15 (68.2%)*	21 (95.5)*	**0.0143**

**Abbreviations:** Post, postoperative; Pre, preoperative; SD, standard deviation.

**Notes:** Values of *p* < 0.05 were deemed significant and written in bold. *Regarding the gap of the fragment, those that were not displaced (wgap < 2mm) were considered within the normal range.

The correlation analysis was performed to assess the effect of the dorsal fragment area on the functional scores (GW and DASH), grip strength percentage, and ROM (flexion, extension, radial deviation, ulnar deviation, pronation, and supination). There was no strong correlation for UC fragment area, LT fragment area, total dorsal fragment area, or fixed area percentage. Although all the UC fragment ratios were higher than 25%, only 4 UC fragments could be fixed by a screw through the volar plate; and the mean area of the ones that were fixed was significantly larger (0.8 ± 0.3 cm ^2^ versus 1.2 ± 0.2 cm ^2^ , *p* = 0.0223). There was no such significant difference regarding LT fragment fixation (0.6 ± 0.4 cm ^2^ versus 1.0 ± 0.8 cm ^2^ ; *p* = 0.1907)

**Fig. 5:**
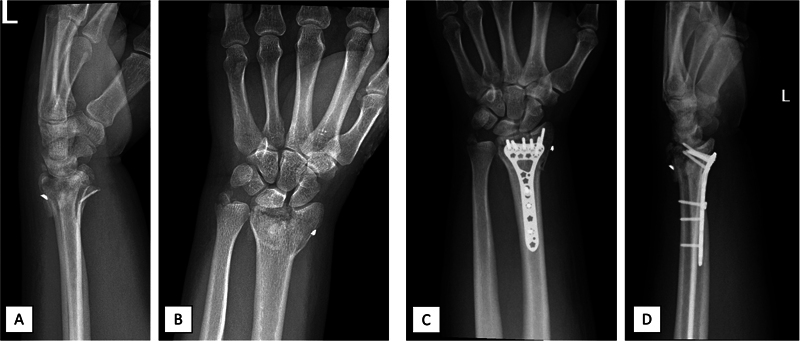
Pre- ( **A,B** ) and postoperative ( **C,D** ) x-rays of patient number 3, who presented an additional ulnar styloid fracture and minimal UC fragment displacement (not fixed with a screw), comminution, and delayed union. The object depicted along the radial styloid is a foreign body from childhood.

**Fig. 6:**
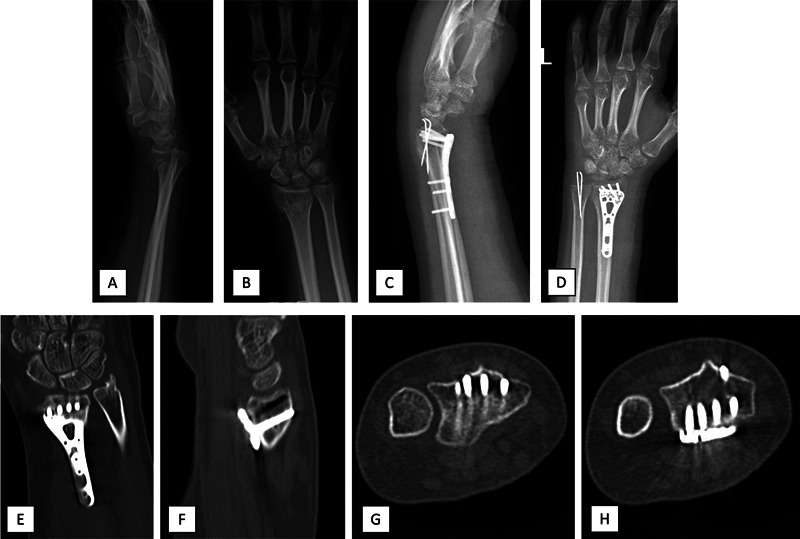
Pre- ( **A,B** ) and postoperative x-rays ( **CD** ) and computed tomography ( **E–H** ) scans of patient number 5, who presented a DRUJ dislocation preoperatively (ulnar styloid base fracture fixed with tension band wires), had no DRUJ subluxation, but had one screw affecting the index finger extensors dorsally.

Out of 22 patients in the current study, 8 presented concurrent wrist or forearm fractures, and 4 of these patients experienced complications. Five patients presented an additional ulnar styloid fracture, 1, a radial head fracture, 1, a scaphoid fracture, and 1, distal radioulnar joint (DRUJ) dislocation. Patient number 3, who presented with an ulnar styloid fracture and comminution, had a minimally displaced UC fragment (2.4 mm postoperatively) that was not fixed with a screw. The patient experienced delayed union ( Fig. 5 ). Patient number 5, who presented a DRUJ dislocation in the form of an ulnar styloid fracture that was fixed with tension band wires, maintained DRUJ stability, but one screw affected the index finger extensors dorsally ( Fig. 6 ). The volar plate in patient number 7 was removed due to irritation and flexor tenosynovitis. Lastly, patient number 10 was a heavy smoker and experienced delayed union.

## Discussion

Despite innovations in orthopedic surgery, treating intra-articular DRFs accompanying dorsal rim fractures is challenging. In a few studies 14 15 16 17 on the treatment of this complex fracture with a standard volar locking plate, dorsal-free fracture fragments could not be fixed adequately but healed well. In the present study, there were no unhealed dorsal fragments, but, in 2 patients, dorsal fragment healing was delayed and took more than 3 months.

All patients in the present study achieved excellent reduction and stability of the UC and LT fragments through fixed-angle volar locking plates. While we anticipated good functional outcomes, success lies in the details. The oversight of dorsal rim fractures during surgery can lead to long-term issues, which prompted the conduct of this study. Therefore, we remained vigilant of complications associated with these fragments and thoroughly elucidated them.

Vanhaecke and Fernandez 18 reported that the success of the procedure using fixed- angle volar plates relies on achieving solid fixation and excellent subchondral bone support. In the current study, we did not experience fixation failure in any patient with fixed-angle volar plates.

With a single volar incision, it is difficult to reduce and secure the fractured dorsal block against the volar bone, but surgeons still try to solve everything on the volar side. 19 To overcome this problem, some surgeons 20 21 have attempted to reduce the displaced broken block by using manual traction or adding a mini dorsal incision. Traction cannot correct the dorsal slope of the fractured distal part because the intact volar radiocarpal ligaments are shorter and thicker than the dorsal radiocarpal ligaments. Thus, longitudinal traction may sometimes adversely affect the reduction of dorsal bone fragments. Due to this mechanical bias, we did not include accompanying volar fractures of the lunate facet in the current study. According to the surgical technique by Orbay, the brachioradialis tendon of the patients was cut from the insertion, the proximal radius was turned down, and the fractured dorsal block and articular surface were entirely exposed. 22 If the dorsal bone block was large enough, the fragment was fixed with a single screw from the holes of the volar plate and drilled only to the near cortex to avoid extensor tendon irritation. Despite all the efforts to reduce and fix dorsal fragments with the volar approach, we do not know how these fragments have healed so far. In the present study, the initial displacement and comminution of the UC fragment did not affect the healing process. If only the volar cortex was reduced without addressing the dorsal fragments, the outcome might not have been as favorable. This is due to the necessity of indirectly manipulating and reducing the dorsal fragments from the volar aspect.

The GW and DASH scores are among the frequently-employed outcome measures outlined in the literature. 23 The DASH score stands out as the choice to assess patients with conditions affecting multiple joints of the upper limb, emphasizing comprehensive upper extremity functionality. 24 On the other hand, the GW score is the best for evaluation after wrist surgery, but it has yet to be validated. 23 25 The GW uses a demerit-point system that entails an objective assessment of wrist function. It is based on the concept that a minimum of 45 ^o^ of dorsiflexion, 30 ^o^ of palmar flexion, 15 ^o^ of ulnar and radial deviation, and 50 ^o^ pronation and supination are normal. 23 In the current study, the GW and DASH scores were correlated (rho = 0.7774; *p* = 0.000). The GW score was also associated with grip strength (rho = -0.8058; *p* = 0.000) but not the DASH score (rho = -0.3559).

Knirk and Jupiter ^5^ have shown that the correct joint congruency significantly determines the consequences of complex articular DRFs. Despite this, loss of reduction in the volar lunate facet has always been reported. Unfortunately, the impact of displaced dorsal rim fractures has attracted little attention. 26 27 Some publications 15 28 29 show that the displaced dorsal margins have no adverse effect on the radiographic and clinical results. In the present study, dorsal rim fragments did not have a negative effect on outcome measures.

The intra-articular fragments of the distal radius are classified as the volar lunate facet, radial column, dorsoulnar (UC), and dorsal rim (LT) fragments. 30 A displaced dorsoulnar fragment is the most common type of fracture in DRFs. 31 The displaced dorsoulnar fragments may also affect DRUJ kinematics; thus, supination is frequently lost in these patients. 32 In the current study, the postoperative reduction of the intermediate column was also adequate, but loss of rotation was observed in 1 case despite a congruent DRUJ. In the present study, we observed that the dorsal bone block containing the LT was healed due to its central location. The present is the first literature report on the surface area properties of this dorsal bone block and its healing process.

Lee et al. 33 studied 48 patients with unstable DRFs, with dorsoulnar fragments with a displacement of more than 2 mm and more than 1/4 of the joint surface. They reported that the effort to fix the dorsoulnar fragment from the volar plate holes did not affect the outcomes. Considering the surface area and volume of this fragment, we could only tightly hold four fragments from the volar side. Additionally, Kim and Cho 17 noted that, in cases in which the dorsal fragment is thin, the lack of fixation does not appear to compromise stability or the clinical outcome. Similarly, in our cases, the fixation of the dorsal fragments with volar screws did not affect the results either.

Although the mechanical relationship between joint surface incongruency and posttraumatic osteoarthritis is still not fully understood, osteoarthritis is believed to be caused by changes in radiocarpal stress. 34 Bradway et al. 35 reported that a > 2 mm step off on the radial joint surface is essential in the development of posttraumatic arthritis. In addition, it has been reported 36 that the function of the carpal joint following DRUJ injury is important for the prognosis, and it affects pain, joint instability, and forearm rotation. Therefore, reduction and fixation of the radial notch should be as crucial as those of the radiocarpal articular surface in treating DRFs. In the current study, none of the patients presented posttraumatic DRUJ arthritis, but the follow-up period was insufficient to evaluate this condition.

Axelrod et al. 37 introduced a limited approach by attempting reduction through a small longitudinal incision of approximately 2 cm on the dorsal side of the distal radius. Thus, the reduction of the radial notch with the posterior incision is also facilitated. In cases in which the dorsal ulnar fragment cannot be reduced volarly, a mini dorsal incision can be used. These dorsal plates should be followed closely, and if they cause synovitis, they must be removed as soon as possible before causing a tendon rupture.

The current study is subject to several limitations that warrant consideration. Firstly, it should be noted that this is a retrospective study focusing on a relatively homogenous patient group. Regrettably, there were no patients with isolated UC or LT fragments, which limited the scope for a comprehensive analysis of these specific conditions. The absence of patients presenting postoperative displacement precludes our ability to comment on the functional ramifications of a displaced dorsal rim fracture. Additionally, no patients were treated with dorsal or double plates through dorsal incisions, thus precluding an assessment of their efficacy and value. Secondly, most participants were of a young age group, with a mean age of 39.7 ± 10.7 years. Consequently, the generalizability of our results to elderly osteoporotic patients might be limited.

## Conclusion

Treating complex intra-articular DRFs with fixed-angle plate fixation through a volar approach yields good early functional results. Healing of the dorsal ulnar fragment towards the volar ulnar fragment is proportional to the gap between these fragments. Distal ulnar screws driven through the volar plate do not always help the dorsal ulnar fragments heal uneventfully. While LT fragments may heal without issues under any condition, there is a potential for long-term problems if the UC fragment is not well reduced and stabilized. This is the key message of the present study. In case of doubt in fluoroscopy, mini dorsal incisions may be helpful, but studies are needed to evaluate their efficacy. In cases of intra-articular dorsal fragmented DRFs with volar plates, there is a possibility of pain and limited rotation of the forearm due to slowly-developing osteoarthritis, and this should not be underestimated.

While the current study provides valuable insights, the limitations should be considered when interpreting and extrapolating the results. Further research encompassing diverse patient groups and prospective designs is necessary to expand the breadth of knowledge in this field.
