# Supplementary material for: Treatment Effectiveness of Volar Plates in Distal Radius Dorsal Rim Fractures
Source: Rev Bras Ortop (Sao Paulo). 2024 Dec 21;59(6):e981–90. doi: 10.1055/s-0044-1790579 (PMC11663069; doi:10.1055/s-0044-1790579)
Supplement: Related texts

# Research article: Eficácia do tratamento com placas volares em fraturas da borda dorsal do rádio distal

**Keywords:** estudos retrospectivos, fixação interna de fraturas, fraturas do rádio, placas ósseas

## Abstract

**Objetivo** Avaliar a eficácia das placas volares do rádio distal em casos de fragmentos dorsais na borda cubital (BC) e no tubérculo de Lister (TL).

**Métodos** Estudo retrospectivo que incluiu pacientes com fraturas do rádio distal (FRDs) com fragmentos dorsais na BC e no TL tratados com placas volares. Foram excluídos pacientes com fraturas da faceta semilunar, razão de fragmentos da BC inferior a 25% e tratamento com placas dorsais. As medidas radiográficas e tomográficas incluíram comprimento radial (CR), inclinação radial (IR), variância cubital (VC), inclinação palmar (IP), áreas de fragmentos, razão de fragmentos da BC e luxações. Foram avaliados as pontuações na classificação de Gartland Werley (GW) e no questionário Disabilities of the Arm, Shoulder and Hand (DASH), além da força de preensão e da amplitude de movimento (ADM).

**Resultados** O estudo incluiu 17 homens e 5 mulheres (idade média: 39,7 ± 10,7 anos). Os fragmentos da BC e do TL apresentaram áreas médias de 1,62 ± 0,73 cm ^2^ e razão de fragmentos da BC de 0,4 ± 0,1. As taxas de fixação dos fragmentos da BC e do TL foram de 18,12% e 31,8%, respectivamente. Melhoras na IR, na VC e na IP foram observadas após a cirurgia. As médias das pontuações na GW e no DASH foram de 2,1 ± 2,0 e 4,3 ± 3,2, respectivamente. A força de preensão no lado operado foi de 89,5 ± 9,8% em comparação ao lado sadio, e pelo menos 90,9% dos pacientes alcançaram ADM adequada.

**Conclusão** Embora as placas volares sejam o tratamento padrão para FRDs intra-articulares, fragmentos dorsais deslocados podem afetar os resultados. Mini-incisões dorsais podem auxiliar na fixação de fragmentos da BC de tratamento difícil com placas volares, para preservar a saúde das articulações.

## Introdução

As fraturas do rádio distal (FRDs) são comuns e geralmente causadas por traumas de alta energia, como quedas acidentais ou acidentes de trânsito. 1 Lesões de alta energia podem provocar FRDs cominutivas intra-articulares, que tendem a incluir fragmentos livres de fraturas luxadas dorsais ou volares. Como essas fraturas estão descritas na classificação de fraturas intra-articulares, requerem redução anatômica e fixação rígida de acordo com os princípios da Associação para o Estudo da Fixação Interna (Arbeitsgemeinschaft für Osteosynthesefragen, AO, em alemão). 2

Com o desenvolvimento da tecnologia de placa bloqueada volar de ângulo fixo, essas fraturas são normalmente fixadas somente por meio de incisão volar. 3 Com essa abordagem, fragmentos livres podem ser facilmente visualizados e reduzidos. Por outro lado, o controle direto dos fragmentos dorsais não é possível. Suponhamos que a fixação de um fragmento dorsal pela abordagem volar não tenha estabilidade suficiente; a fratura pode sofrer luxação, o que leva à consolidação viciosa ou não consolidação, bem como artrite pós-traumática e piora da função do punho. 4 5 Se os fragmentos dorsais livres forem grandes o suficiente, são fixados com os parafusos nos orifícios distais da placa volar. No entanto, nem sempre é possível avaliar se esses parafusos fixam bem os fragmentos dorsais por meio de fluoroscopia no centro cirúrgico.

A técnica da placa dorsal também foi descrita como método de fixação de fragmentos intra-articulares dorsais instáveis. No entanto, foram observadas complicações graves na placa dorsal, incluindo irritação e ruptura do tendão. 6 Uma abordagem palmar e dorsal combinada é eficaz para a redução e fixação, mas afeta negativamente o rádio distal e a vascularização cutânea, ao causar mais trauma em tecidos moles. 7

O objetivo principal deste estudo foi avaliar a eficácia da placa volar para a fixação de fraturas da borda dorsal do rádio distal em termos da promoção da redução e estabilização dos fragmentos da borda cubital (BC) e do tubérculo de Lister (TL).

## Materiais e Métodos

Este estudo foi aprovado pelo comitê de ética institucional (sob o número E1-23-3412).Trata-se de um estudo de coorte retrospectivo de FRDs com fragmentos dorsais. Os pacientes com FRD operados entre maio de 2019 e outubro de 2021 foram identificados nos prontuários hospitalares. Pacientes com FRDs sem fragmentos dorsais, fraturas dos tipos 23A ou B da AO, aqueles atendidos 5 semanas depois do evento, com fraturas da faceta semilunar volar, operados com placas dorsais, e aqueles com fragmentos somente na BC ou no TL foram excluídos. Em nossa clínica, como as FRDs eram de tipo C da AO, radiografias e tomografias computadorizadas (TCs) de rotina foram realizadas no pós-operatório, com ênfase especial no monitoramento de complicações em longo prazo, e alguns casos foram submetidos a intervenções. Também foram excluídos pacientes perdidos durante o acompanhamento e aqueles sem radiografias ou TCs, além daqueles com razão de fragmentos da BC inferior a 25%, menores de 18 anos, com doenças neuromusculares e com lesões contralaterais nos membros superiores. Assim, este estudo incluiu 22 pacientes com FRDs intra-articulares com fragmentos de BC e TL submetidos ao tratamento com placas volares. O processo de inclusão e exclusão de pacientes está detalhado na Fig. 1 .

**Fig. 1:** Processo de inclusão e exclusão de pacientes.

Todas as cirurgias foram realizadas pelos dois cirurgiões ortopédicos especialistas em mão (UB e EA), com experiência cirúrgica de níveis 3 a 4, conforme o estudo de Tang e Giddins. 8 Usando uma abordagem volar e pronando o eixo do rádio, o grande fragmento dorsal foi temporariamente fixado com um fio de Kirschner. Um parafuso foi então usado para a fixação sobre a placa, garantindo que ela não penetrasse no córtex dorsal para evitar a ruptura do tendão extensor. Além disso, foi tomado cuidado para garantir que a placa não cruzasse a linha divisória, evitando a ruptura do tendão flexor. Caso a fixação temporária garantisse a fixação do parafuso da placa, o fio de Kirschner era removido, e se a fixação continuasse estável, a cirurgia era encerrada. O “truque” mais importante no uso da placa volar para a fixação do fragmento dorsal da BC examinado é a fixação das BCs volares e dorsais com uma pinça Weber larga, seguida de perfuração sob orientação fluoroscópica ( Fig. 2 ). A compreensão completa da anatomia da incisura radial é necessária para evitar perfuração articular. Se o fragmento for muito pequeno para ser fixado com parafuso, o polegar e o indicador da mão esquerda podem atuar como a pinça Weber durante a perfuração, alinhando os fragmentos e permitindo a colocação cuidadosa do parafuso sobre a placa. Durante a cirurgia, uma boa radiografia *skyline* à fluoroscopia não apenas revela a relação do parafuso mais cubital com o córtex dorsal, como também permite a visualização de sua relação com a incisura radial. Usamos essa projeção tangencial principalmente para visualizar a incisura radial à cirurgia porque nunca penetramos o córtex dorsal. A realização desta técnica pode não ser simples para um cirurgião com níveis 1 a 2 de experiência.

**Fig. 2:** O “truque” para usar a placa volar para a fixação do fragmento dorsal da borda cubital. Fixação da placa com o polegar na placa e o indicador no dorso do punho ( **A** ). Fixação do fragmento da borda cubital dorsal com pinça Weber larga ( **B,C** ). Perfuração sob orientação fluoroscópica ( **D** ). Controle dos parafusos para a redução da incisura radial e a penetração indesejada do parafuso da articulação radiocubital distal com visualização *skyline* à fluoroscopia ( **E** ).

Depois da identificação dos pacientes nos prontuários hospitalares, analisamos seus estudos radiológicos pré e pós-operatórios, as radiografias diretas e as TCs. Como parâmetros radiológicos, medimos a inclinação radial (IR), a altura radial (AR), a variância cubital (VC) e a inclinação palmar (IP) em radiografias pré e pós-operatórias do lado acometido. As radiografias pós-operatórias foram obtidas às 6 semanas, 3 meses, 6 meses e 1 ano, e, depois, 1 vez ao ano. Os valores do primeiro e do último raio-x dos pacientes foram avaliados. Todas as medidas foram feitas pelo cirurgião sênior (UB). Os critérios de Jupiter 9 10 foram utilizados para as variáveis radiológicas. Os critérios de não aceitação foram IR < 10 ^o^ , inclinação volar > 20 ^o^ ou inclinação dorsal > 20 ^o^ , AR < 10 mm, VC > 2 mm e incongruência ou intervalo intra-articular > 2 mm. 9 10 As TCs foram usadas para avaliações distintas da largura e do comprimento dos fragmentos dorsais da BC e do TL e o cálculo da área superficial. A área total do fragmento dorsal é a soma dos fragmentos da BC e do TL. Além disso, a razão entre a profundidade do fragmento da BC e a profundidade do rádio distal foi calculada ( Fig. 3 ). Registramos se o fragmento foi fixado ou não com o parafuso da placa volar ( Fig. 4 ). Também calculamos o percentual de área fixada, correspondente ao percentual da área do fragmento dorsal fixado com parafuso.

**Fig. 3:** Ilustração dos fragmentos dorsais ( **A** ). Medida da razão de fragmentos da borda cubital (BC) ( **B** ). **Abreviatura:** TL: tubérculo de Lister.

**Fig. 4:** Neste exemplo, o fragmento dorsal foi fixado com parafuso ( **A** ). Neste outro exemplo, o fragmento dorsal não pôde ser fixado com parafuso. A união foi obtida pelo lado radial ( **B** ).

Os pacientes passaram por uma consulta de acompanhamento para avaliação funcional, que incluiu as pontuações na classificação de Gartland Werley (GW) e no questionário Disabilities of the Arm, Shoulder and Hand (DASH), e as medida da força de preensão com um dinamômetro e da amplitude de movimento (ADM) com um goniômetro. Um cirurgião júnior calculou todas as pontuações, e um cirurgião sênior realizou as medições. Os valores máximos normais de ADM foram de 70° de extensão, 75° de flexão, 20° de desvio radial, 35° de desvio cubital, 70° de pronação e 85° de supinação. 11 Segundo Ryu et al., 12 a maioria das tarefas funcionais pode ser realizada com 70% por cento da ADM máxima do punho; logo, os valores mínimos aceitos foram de 49° de extensão, 52,5° de flexão, 14° de desvio radial, 24,5° de desvio cubital, 49° de pronação e 59,5° de supinação. A força de preensão foi medida tanto no lado acometido quanto no lado saudável, e a sua porcentagem no lado acometido em comparação ao lado saudável foi calculada para análise posterior.

Realizamos análises descritivas e inferenciais. O teste de Shapiro-Wilk foi utilizado para a avaliação da normalidade. Os testes do Qui-quadrado e exato de Fisher foram usados para as variáveis categóricas. Usou-se um teste *t* para a análise dos dados paramétricos dos grupos. O teste U de Mann-Whitney foi realizado para analisar os dados não paramétricos dos grupos. Um teste *t* pareado foi usado para comparar dois valores de grupos independentes. Valores de *p* inferiores a 0,05 foram considerados significativos. A análise de correlação usou o teste de Pearson se ambas as variáveis fossem paramétricas, e o teste de Spearman caso alguma das variáveis não fosse paramétrica. O valor de rho ≥ 0,8 foi aceito como indicativo e correlação forte, de acordo com Chan. 13

## Resultados

Os dados demográficos, as dimensões dos fragmentos, as pontuações funcionais e a ADM estão resumidos na Tabela 1 . O período médio de acompanhamento foi de 24,1 ± 4,1 (mínimo: 17; máximo: 32) meses. No último acompanhamento, os formulários de pesquisa foram preenchidos e as pontuações funcionais foram calculadas. A média de força de preensão do lado operado foi de 35,0 ± 6,0 kg e, do lado sadio, de 39,1 ± 4,6 kg, com diferença significativa ( *p* = 0,0002). A porcentagem média do lado operado em relação ao lado sadio foi de 89,5 ± 9,8%. A ADM mínima necessária foi alcançada em 90,9% para flexão, extensão, desvio radial e desvio ulnar, enquanto todos os pacientes (100%) atingiram faixa normal para pronaç ão e supinação.

**Tabela 1:** Dados demográficos, do fragmento dorsal e funcionais da amostra do estudo

**Idade (anos): média ± DP**	**39,7 ± 10,7**
**Gênero: n (%)** ** Feminino** ** Masculino**	5 (22,7) 17 (77,3)
**Lado: n (%)** ** Direito** ** Esquerdo** **Dominância: n (%)**	11 (50,0) 11 (50,0) 11 (50,0)
**Tempo até a apresentação (dias): média ± DP**	15,6 ± 11,3
**Tempo de acompanhamento (meses): média ± DP**	24,1 ± 4,1
**Borda cubital** ** Largura (mm): média ± DP** ** Profundidade (mm): média ± DP** ** Área (cm ^2^ ): média ± DP ** ** Fixação com parafuso do fragmento: ^a^ n (%) ** ** Razão de fragmentos da BC: média ± DP** ** Lacuna pré-operatória (mm): média ± DP** ** Lacuna pós-operatória (mm): média ± DP**	13,4 ± 3,1 6,6 ± 1,4 0,9 ± 0,3 4 (18,2) 0,4 ± 0,1 2,0 ± 1,2 0,2 ± 0,6
**Tubérculo de Lister** ** Largura (mm): média ± DP** ** Profundidade (mm): média ± DP** ** Área (cm ^2^ ): média ± DP ** ** Fixação com parafuso do fragmento: ^a^ n (%) **	9,3 ± 3,00 7,3 ± 3,2 0,7 ± 0,5 7 (31,8)
** Área total do fragmento (cm ^2^ ): média ± DP ** **Área do fragmento fixado (%)**	1,6 ± 0,7 44,5 ± 41,3
**Pontuação na classificação de Gartland Werley: média ± DP**	2,1 ± 2,0
**Pontuação no DASH: média ± DP**	4,3 ± 3,2
**Força de apreensão (kg): média ± DP** ** Lado operado** ** Lado saudável (controle)** **Operado/Controle (%)**	35,0 ± 6,0 39,1 ± 4,6 89,5 ± 9,8
** Amplitude de movimento: média ± DP/n (%) ^b^** ** Extensão** ** Flexão** ** Desvio radial** ** Desvio cubital** ** Pronação** ** Supinação**	69,6 ± 9,3/20 (90,9%) 70,5 ± 10,3/20 (90,9%) 21,1 ± 4,4/20 (90,9%) 28,5 ± 5,9/20 (90,9%) 73,4 ± 6,6/22 (100%) 78,5 ± 8,3/22 (100%)

**Abreviaturas:** DASH, questionário *Disabilities of the Arm, Shoulder and Hand* ; DP, desvio padrão; GW, classficação de Gartland Werley.

**Notas:** Para as variáveis categóricas, “n” é o número de pacientes que atendem à condição, e “%” é o percentual desses pacientes. ^a^ A fixação do fragmento com parafuso indica a presença de um parafuso que segura o fragmento. ^b^ Neste contexto, “n” é o número de pacientes capazes de atingir a amplitude mínima necessária para cada movimento específico, e “%” é a porcentagem desses pacientes.

A Tabela 2 mostra a comparação dos parâmetros radiológicos nos períodos pré e pós-operatório. A AR, a VC e a IP melhoraram significativamente após a cirurgia. Todos os fragmentos dorsais apresentavam luxação e foram reduzidos com sucesso com a abordagem cirúrgica volar pela técnica de Orbay. A fixação foi feita com o parafuso da placa volar em quatro pacientes no fragmento da BC e em sete pacientes no fragmento do TL. Apesar disso, houve uma luxação da BC de 2,4 mm em um paciente que não cumpriu os protocolos de acompanhamento. Além disso, pelo menos 81,82% dos pacientes estavam dentro da normalidade em relação a todos os parâmetros.

**Tabela 2:** Parâmetros pré e pós-operatórios da amostra do estudo

Parâmetro	Pré-operatório: média ± DP	Pós-operatório: média ± DP	*p*	Diferença (pós e pré): média ± DP	Amplitude normal pré-operatória: n (%)	Amplitude normal pós-operatória: n (%)	*p*
**Inclinação radial**	22,6 ± 5,1	23,2 ± 3,1	0,5151	0,6 ± 4,4	22 (100%)	22 (100%)	1,000
**Altura radial**	11,8 ± 3,6	13,1 ± 2,4	**0,0016**	1,3 ± 1,5	18 (81,8%)	20 (90,9%)	0,1573
**Variância cubital**	1,3 ± 1,6	0,1 ± 1,6	**0,0124**	-1,2 ± 2,0	10 (45,5%)	18 (81,8%)	**0,0047**
**Inclinação palmar**	-4,3 ± 15,8	3,8 ± 3,7	**0,0457**	9,1 ± 16,1	18 (81,8%)	22 (100%)	**0,0455**
**Lacuna do fragmento**	2,0 ± 1,2	0,2 ± 0,6	**0,0000**	1,8 ± 0,8	15 (68,2%)*	21 (95,5)*	**0,0143**

**Abreviaturas:** DP, desvio padrão; Pós, pós-operatório; Pré, pré-operatório.

**Notas:** Val.ores de *p* < 0,05 foram considerados significativos e escritos em negrito. *Em relação à lacuna do fragmento, aqueles sem luxação (com lacuna < 2 mm) foram considerados dentro da faixa de normalidade.

Por meio da análise de correlação, verificou-se o efeito da área do fragmento dorsal nas pontuações funcionais (GW e DASH), no percentual de força de preensão e na ADM (flexão, extensão, desvio radial, desvio cubital, pronação, supinação). Não houve correlação forte com a área de fragmentos da BC, a área de fragmentos do TL, a área total do fragmento dorsal ou a porcentagem de área fixada. Embora todas as proporções da BC fossem superiores a 25%, apenas 4 fragmentos da BC puderam ser fixados por parafuso com placa volar; além disso, a média da área dos fragmentos fixados foi significativamente maior (0,8 ± 0,3 cm ^2^ *versus* 1,2 ± 0,2 cm ^2^ ; *p* = 0,0223). Não houve diferença significativa quanto à fixação do fragmento do TL (0,6 ± 0,4 cm ^2^ *versus* 1,0 ± 0,8 cm ^2^ ; *p* = 0,1907).

Dos 22 pacientes do estudo, 8 apresentavam fraturas concomitantes no punho ou no antebraço, e 4 deles tiveram complicações. Cinco pacientes tiveram uma fratura estiloide cubital, um apresentou uma fratura na cabeça do rádio, um teve uma fratura do escafoide e um apresentou luxação na articulação radiocubital distal (ARCD). O paciente de número 3, que tinha uma fratura estiloide cubital, apresentou luxação mínima do fragmento da BC (de 2,4 mm após a cirurgia) que não foi fixada com parafuso. O paciente sofreu consolidação tardia ( Fig. 5 ). O paciente de número 5, que apresentava uma luxação da ARCD na forma de uma fratura estiloide cubital que foi fixada com fios de banda de tensão, manteve a estabilidade desta articulação; porém, um dos parafusos afetava os extensores do dedo indicador dorsalmente ( Fig. 6 ). A placa volar do paciente de número 7 foi removida devido à irritação e tenossinovite dos flexores. Por último, o paciente de número 10 era fumante inveterado e apresentou consolidação tardia.

**Fig. 5:** Radiografias pré-operatórias ( **A,B** ) e pós-operatórias ( **C,D** ) do paciente de número 3, que apresentava fratura estiloide cubital, luxação mínima do fragmento da borda cubital (não fixado com parafuso), cominuição e consolidação tardia. O objeto representado ao longo do estiloide radial é um corpo estranho presente desde a infância.

**Fig. 6:** Radiografias pré-operatórias ( **AB** ), radiografias pós-operatórias ( **C,D** ) e tomografia computadorizada ( **E–H** ) do paciente de número 5, com luxação pré-operatória da articulação radiocubital distal (ARCD) (fratura da base estiloide do cúbito fixada com fios de banda de tensão). O paciente não apresentava subluxação da ARCD, mas tinha um parafuso que afetava dorsalmente os extensores do dedo indicador.

## Discussão

Apesar das inovações na cirurgia ortopédica, o tratamento das fraturas intra-articulares do rádio distal que acompanham as fraturas da borda dorsal é desafiante. Em alguns estudos 14 15 16 17 acerca do tratamento dessa fratura complexa com uma placa de bloqueio volar padrão, os fragmentos de fratura livre dorsal não puderam ser fixados adequadamente, mas cicatrizaram bem. Neste estudo, não houve ausência de consolidação dos fragmentos dorsais, mas dois pacientes apresentaram consolidação tardia dos fragmentos dorsais, um processo que demorou mais de três meses.

Todos os pacientes do estudo obtiveram excelente redução e estabilidade dos fragmentos da BU e do TL por meio de placas bloqueadas volares de ângulo fixo. Embora tenhamos antecipado bons desfechos funcionais, o sucesso jaz nos detalhes. A negligência de fraturas da borda dorsal durante a cirurgia pode levar a problemas de longo prazo, o que motivou a realização deste estudo. Portanto, continuamos vigilantes quanto às complicações associadas a esses fragmentos e os elucidamos minuciosamente.

Vanhaecke e Fernandez 18 relataram que o sucesso do procedimento que usa a placa volar de ângulo fixo depende da obtenção de fixação sólida e excelente apoio ósseo subcondral. Neste estudo, não houve casos de falha de fixação em nenhum paciente tratado com placas volares de ângulo fixo.

É difícil reduzir e fixar o bloco dorsal fraturado contra o osso volar com apenas uma incisão volar, mas os cirurgiões ainda tentam resolver tudo do lado volar. 19 Para superar esse problema, alguns cirurgiões 20 21 tentaram reduzir o bloco fraturado e luxado por tração manual ou com o acréscimo de uma mini-incisão dorsal. A tração não pode corrigir a inclinação dorsal da parte distal fraturada porque os ligamentos radiocarpais volares intactos são mais curtos e grossos do que os ligamentos radiocarpais dorsais. Assim, a tração longitudinal às vezes pode prejudicar a redução dos fragmentos ósseos dorsais. Devido a esse viés mecânico, não incluímos fraturas da faceta semilunar volar neste estudo. De acordo com a técnica cirúrgica de Orbay, o tendão braquiorradial dos pacientes foi seccionado a partir de sua inserção, o rádio proximal foi virado para baixo, e o bloco da fratura dorsal e a superfície articular foram expostos por completo. 22 Se o bloco ósseo dorsal fosse grande o suficiente, o fragmento era fixado com um único parafuso a partir dos orifícios da placa volar e perfurado apenas até a cortical próxima, para evitar irritação do tendão extensor. Apesar de todos os esforços para reduzir e fixar os fragmentos dorsais com a abordagem volar, não sabemos como esses fragmentos se consolidaram até o momento. Neste estudo, a luxação inicial e a cominuição do fragmento da BC não afetaram o processo de consolidação. Se apenas o córtex volar fosse reduzido sem abordar os fragmentos dorsais, o resultado poderia não ter sido tão favorável. Isto se deve à necessidade de manipular e reduzir indiretamente os fragmentos dorsais da face volar.

As pontuações na GW e no DASH estão entre as medidas de desfecho mais empregadas na literatura. 23 A pontuação no DASH destaca-se como a preferida para a avaliação de pacientes com acometimento de múltiplas articulações dos membros superiores, o que enfatiza a sua funcionalidade abrangente. 24 Por outro lado, a pontuação na GW é a melhor para a avaliação após a cirurgia do punho, mas ainda precisa ser validada. 23 25 A classificação de GW utiliza um sistema de pontos de demérito que requer uma avaliação objetiva da função do punho. Baseia-se no conceito de que um mínimo de 45° de dorsiflexão, 30° de flexão palmar, 15° de desvio cubital e radial e 50° de pronação e supinação são normais. 23 neste estudo, as pontuações na GW e no DASH apresentaram correlação (rho = 0,7774; *p* = 0,000). A GW também foi associada à força de preensão (rho = - 0,8058; *p* = 0,000), mas não o DASH (rho = -0,3559).

Knirk e Jupiter ^5^ demonstraram que a congruência articular correta determina significativamente os desfechos de FRDs articulares complexas. Apesar disso, a perda da redução da faceta semilunar volar foi sempre relatada. Infelizmente, o impacto das fraturas com luxação da borda dorsal tem atraído pouca atenção. 26 27 Algumas publicações 15 28 29 mostram que as margens dorsais luxadas não têm efeito adverso nos desfechos radiográficos e clínicos. Neste estudo, os fragmentos da borda dorsal não tiveram um efeito negativo nas medidas de desfechos.

Os fragmentos intra-articulares do rádio distal são classificados como faceta volar semilunar, coluna radial, fragmentos dorsocubitais (BC) e borda dorsal (TL). 30 A luxação do fragmento dorsocubital é o tipo mais comum de FRD. 31 Os fragmentos dorsocubitais luxados também podem afetar a cinemática da ARCD; por isso, estes pacientes tendem a perder a capacidade de supinação. 32 Neste estudo, a redução intermediária da coluna no pós-operatório também foi adequada, mas houve perda de rotação em um caso, apesar da congruência da ARCD. Neste estudo, observamos que o bloco ósseo dorsal que contém o TL estava cicatrizado devido à sua localização central. Este é o primeiro relato na literatura das propriedades da área superficial deste bloco ósseo dorsal e seu processo de consolidação.

Lee et al. 33 estudaram 48 pacientes com FRDs instáveis, fragmentos dorsocubitais com luxação de mais de 2 mm e mais de 1/4 da superfície articular. Estes autores relataram que o esforço para a fixação do fragmento dorsocubital nos orifícios da placa volar não afetou os desfechos. Considerando a área superficial e o volume deste fragmento, poderíamos fixar com firmeza somente quatro fragmentos do lado volar. Além disso, Kim e Cho 17 observaram que, nos casos em que o fragmento dorsal é delgado, a ausência de fixação não parece comprometer a estabilidade ou o desfecho clínico. Da mesma forma, em nossos casos, a fixaç ão dos fragmentos dorsais com parafusos volares também não afetou os resultados.

Embora a relação mecânica entre a incongruência da superfície articular e a osteoartrite pós-traumática ainda não seja compreendida por completo, acredita-se que a osteoartrite seja causada por alterações no estresse radiocárpico. 34 Bradway et al. 35 relataram que uma incongruência > 2 mm na superfície articular radial é essencial para o desenvolvimento da artrite pós-traumática. Além disso, foi relatado 36 que a função da articulação do carpo após o acometimento da ARCD é importante no prognóstico e influencia a dor, a instabilidade articular e a rotação do antebraço. Portanto, a redução e a fixação da incisura radial devem ser tão cruciais quanto às da superfície articular radiocarpal no tratamento da FRD. Neste estudo, nenhum paciente apresentou artrite pós-traumática da ARCD, mas o período de acompanhamento foi insuficiente para tal avaliação.

Axelrod et al. 37 introduziram uma abordagem limitada na tentativa de redução com uma pequena incisão longitudinal de aproximadamente 2 cm na face dorsal do rádio distal. Assim, a redução da incisura radial com a incisão posterior também é facilitada. Nos casos em que o fragmento cubital dorsal não pode ser reduzido volarmente, uma mini-incisão dorsal pode ser utilizada. Essas placas dorsais devem ser monitoradas com cuidado e, caso provoquem sinovite, devem ser removidas o mais rápido possível, antes que ocorra a ruptura do tendão.

Este estudo tem várias limitações que merecem consideração. Em primeiro lugar, deve-se notar que se trata de um estudo retrospectivo centrado em um grupo de pacientes relativamente homogêneo. Lamentavelmente, não houve pacientes com fragmentos isolados de BC ou TL, o que limitou o escopo para uma análise abrangente dessas condições específicas. A ausência de pacientes com luxação pós-operatória impediu nossa capacidade de comentar as ramificações funcionais de uma fratura luxada da borda dorsal. Além disso, nenhum paciente foi tratado com placas dorsais ou duplas por meio de incisões dorsais, o que impossibilitou uma avaliação de sua eficácia e valor. Em segundo lugar, a maioria dos participantes do estudo era jovem, com média de idade de 39,7 ± 10,7 anos. Consequentemente, a generalização dos nossos resultados para pacientes idosos com osteoporose pode ser limitada.

## Conclusão

O tratamento de fraturas intra-articulares complexas do rádio distal com fixação usando placa de ângulo fixo e abordagem volar apresenta bons desfechos funcionais iniciais. A consolidação do fragmento cubital dorsal em direção ao fragmento cubital volar é proporcional à lacuna entre esses fragmentos. Parafusos cubitais distais inseridos através da placa volar nem sempre ajudam na consolidação dos fragmentos cubitais dorsais sem intercorrências. Embora os fragmentos do TL possam cicatrizar sem problemas em qualquer condição, há a possibilidade de problemas em longo prazo se o fragmento da BC não estiver bem reduzido e estabilizado. Esta é a mensagem principal deste estudo. Em caso de dúvida na fluoroscopia, mini-incisões dorsais podem ser úteis, porém, mais estudos são necessários para avaliar sua eficácia. O tratamento de fraturas intra-articulares do rádio distal com fragmentos dorsais com placas volares pode causar dor e limitação da rotação do antebraço devido a uma osteoartrite de desenvolvimento lento; este fato não deve ser subestimado.

Embora este estudo traga informações valiosas, as limitações devem ser consideradas ao se interpretar e extrapolar os resultados. Mais pesquisas com grupos diversos de pacientes e projetos prospectivos são necessárias para expandir a amplitude do conhecimento neste campo.
